# Supplementary figures and images for: Sequence Similarity Network Analysis Provides Insight into the Temporal and Geographical Distribution of Mutations in SARS-CoV-2 Spike Protein
Source: Viruses. 2022 Jul 29;14(8):1672. doi: 10.3390/v14081672 (PMC9413517; doi:10.3390/v14081672)

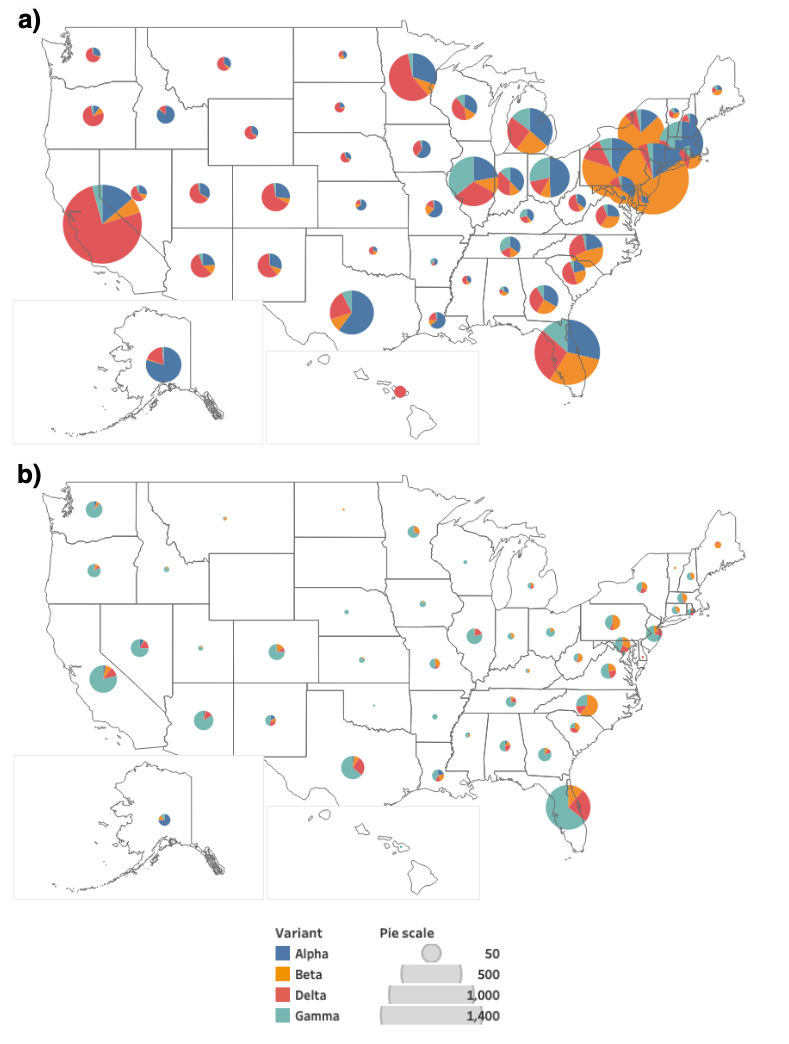

Supplement: Supplementary file 1 [file viruses-14-01672-s001.zip › SupplementaryFigure S1.png]

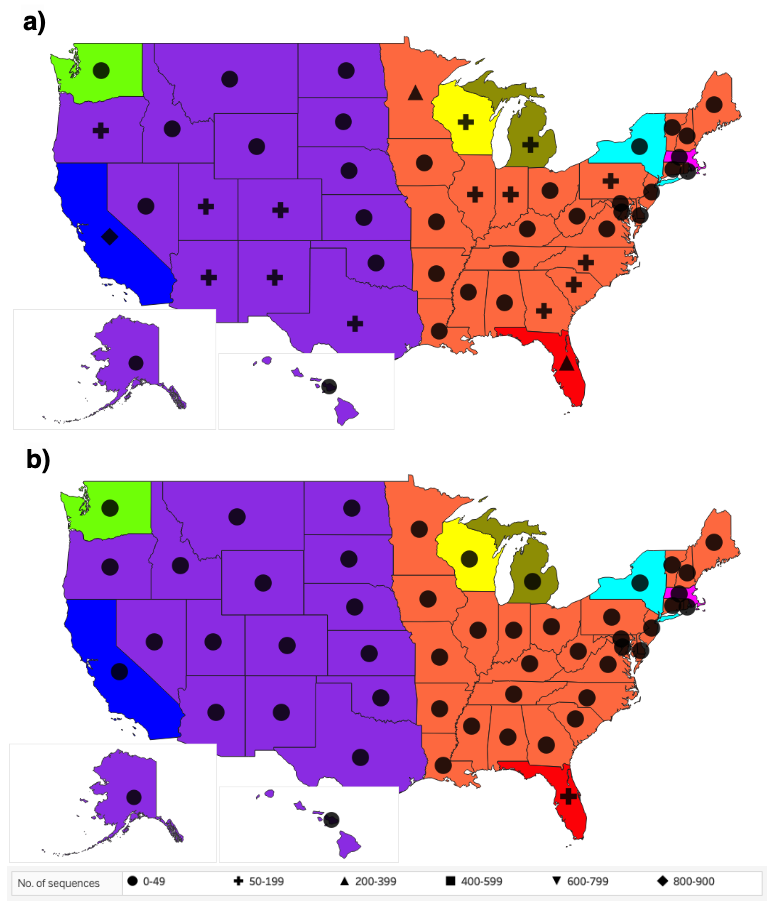

Supplement: Supplementary file 1 [file viruses-14-01672-s001.zip › SupplementaryFigure S2.png]
